# The Link between Prostanoids and Cardiovascular Diseases

**DOI:** 10.3390/ijms24044193

**Published:** 2023-02-20

**Authors:** Livia Beccacece, Paolo Abondio, Carla Bini, Susi Pelotti, Donata Luiselli

**Affiliations:** 1 Computational Genomics Lab, Department of Pharmacy and Biotechnology, University of Bologna, 40126 Bologna, Italy; 2 aDNA Lab, Department of Cultural Heritage, University of Bologna, Ravenna Campus, 48121 Ravenna, Italy; 3 Unit of Legal Medicine, Department of Medical and Surgical Sciences, University of Bologna, 40126 Bologna, Italy

**Keywords:** arachidonic acid pathway, prostanoids, cardiovascular diseases, inflammation, genetic polymorphisms

## Abstract

Cardiovascular diseases are the leading cause of global deaths, and many risk factors contribute to their pathogenesis. In this context, prostanoids, which derive from arachidonic acid, have attracted attention for their involvement in cardiovascular homeostasis and inflammatory processes. Prostanoids are the target of several drugs, but it has been shown that some of them increase the risk of thrombosis. Overall, many studies have shown that prostanoids are tightly associated with cardiovascular diseases and that several polymorphisms in genes involved in their synthesis and function increase the risk of developing these pathologies. In this review, we focus on molecular mechanisms linking prostanoids to cardiovascular diseases and we provide an overview of genetic polymorphisms that increase the risk for cardiovascular disease.

## 1. Introduction

Cardiovascular diseases (CVDs) are the main cause of global mortality, and their global burden is continuously increasing in many countries, as reported by the Global Burden of Disease study. Overall, incidence and mortality rates are higher in men ranging in age from 30–60 years old and in elders. Nevertheless, young people are also affected by cardiovascular diseases [1].

Cardiovascular disease is a comprehensive term that refers to several pathologies affecting the cardiovascular system, such as coronary heart disease, myocardial infarction, stroke, atherosclerosis, cardiomyopathy, and many others [2].

There are many risk factors contributing to the pathogenesis of these diseases; some of these are related to personal behaviors, such as smoking, diet, physical activity, and alcohol consumption, and others are due to molecular factors. Furthermore, there are also pathological conditions, such as diabetes and obesity, that increase the likelihood of developing some cardiovascular diseases. On the whole, several factors may contribute to the development of these pathologies, either alone or together [3].

Relating to molecular factors, there are metabolic processes that have been associated with cardiovascular diseases because some metabolites play a pathogenic role, which can increase or sometimes reduce the risk of developing these pathologies. In these processes, a central role is played by the enzymes involved in these metabolic processes and by genetic variants influencing their activity. Among these metabolic processes, the arachidonic acid pathway plays a crucial role in cardiovascular homeostasis and inflammation and is widely related to several cardiovascular pathologies [4].

## 2. Arachidonic Acid Pathway

**Figure 1:**
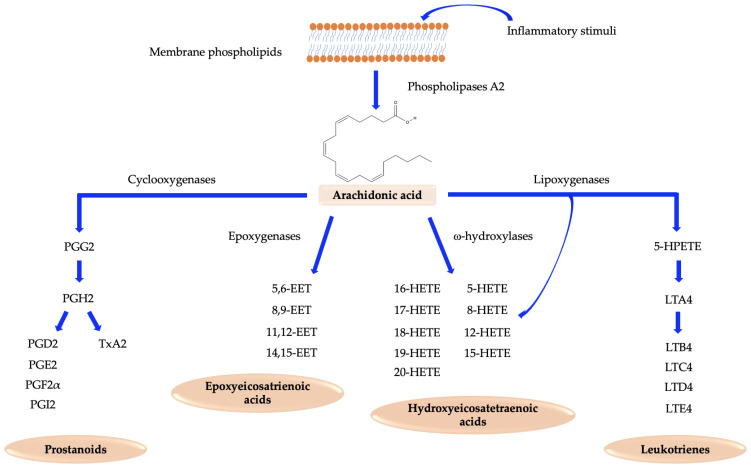
Arachidonic acid metabolism. An overview of the AA pathway, starting from its cleavage from cellular membranes via the phospholipase A2 enzymes, which leads to four classes of eicosanoids: prostanoids, epoxyeicosatrienoic acids, hydroxyeicosatetraenoic acids, and leukotrienes. Eicosanoids are synthesized by three classes of enzymes: cyclooxygenases, lipoxygenases, and CYP450 enzymes, which includes both ω-hydroxylases and epoxygenases.

Arachidonic acid (AA) is a ω-6 polyunsaturated fatty acid (PUFA) that is abundant in cell membrane phospholipids and is mainly obtained through the diet [4]. AA can be metabolized by three different classes of enzymes: cyclooxygenases, lipoxygenases, and cytochrome P450 enzymes (epoxygenases and ω-hydroxylases); these enzymes produce eicosanoids, a series of bioactive lipids (Figure 1). Eicosanoids include prostanoids, leukotrienes, epoxyeicosatrienoic (EETs), and hydroxyeicosatetraenoic acids (HETEs), which play an important role in cardiovascular diseases and inflammatory processes but also in cardiac protection [4,5].

Prostanoids, including prostaglandins and thromboxane A2, are produced by cyclooxygenases (*COX* genes), while leukotrienes are produced by lipoxygenase (*ALOX* genes) activity. They are deeply involved in cardiovascular pathological states, mainly associated with inflammation, thrombosis, and atherosclerosis [6,7,8,9,10]. HETEs are synthesized from arachidonic acid by both lipoxygenases and CYP450 ω-hydroxylases. In addition to prostanoids and leukotrienes, these acids have been related to several pathological states and have pro-inflammatory and cardiotoxic activities. Among these, 20-HETE is the most studied [5,11,12]. EETs are produced only by CYP450 epoxygenases. It can be stated that they differ from other eicosanoids because they exert cardiovascular protective and anti-inflammatory roles [5,13].

The AA pathway and eicosanoids have been studied extensively. This review will focus only on prostanoids and their association with cardiovascular disease and also cover their critical role in inflammatory responses and cardiovascular homeostasis.

## 3. Bioavailability of Arachidonic Acid and PLA2 Enzymes

The bioavailability of arachidonic acid is tightly linked to phospholipase A2 (PLA2) activity that cleaves the AA from cell membranes. This is the first reaction of AA metabolism, and it is the rate-limiting step, influencing the subsequent synthesis of eicosanoids and the risk of cardiovascular disease. The PLA2 enzyme superfamily includes several families of phospholipase A2, categorized based on their localization, function, and dependence on Ca^2+^. Arachidonic acid is hydrolyzed from membrane phospholipids mainly by secretory and cytosolic PLA2s, which in turn can have low substrate specificity or a preference for PUFAs. Generally, cytosolic PLA2s have greater specificity for arachidonic acid. Owing to their role, some PLA2s have been associated with cardiovascular diseases [14,15].

### 3.1. Cytosolic PLA2s

#### 3.1.1. Cytosolic PLA2α

Among PLA2 enzymes, the cytosolic PLA2α, encoded by the *PLA2G4A* gene, is well characterized and plays a central role in the release of AA, showing a strong substrate specificity for this PUFA [14]. This enzyme is activated by different molecules, such as pro-inflammatory interleukin-1α (IL-1α) [16] and tumor necrosis factor α (TNF-α), both of which are produced in response to myocardial ischemia, and contributes to myocardial infarction and reperfusion injury [17]. However, there are conflicting and opposing data on the association of cPLA2α with myocardial ischemia. On one hand, an in vivo study on cPLA2α^−/−^ knockout mice has shown that the absence of this enzyme increases infarction size and reperfusion injury after myocardial ischemia [18]. On the other hand, another study on knockout mice has displayed that inhibition of cPLA2α decreases the myocardial infarct size and preserves left ventricle function, attenuating myocardial ischemia and reperfusion injury owing to a lower cleavage of arachidonic acid and the subsequent synthesis of pro-inflammatory and cardiotoxic eicosanoids [19].

Coming back to human studies, cPLA2α seems to be involved in different cardiovascular pathologies, and there are single nucleotide polymorphisms (SNPs) associated with greater or lower risk of developing them. For instance, it is highly expressed in atherosclerotic lesions, increasing the inflammatory state and foam cell formation [20], and recently it has been found that cPLA2α overexpression is involved in aortic valve calcification, leading to aortic stenosis and other cardiovascular diseases [21].

Regarding polymorphisms in *PLA2G4A*, intronic variant rs12746200 has been identified as a protective variant against CVD development; in particular, it is the derived G allele that reduces the risk of cardiovascular diseases because people carrying GG or AG genotypes show a lower likelihood of major adverse cardiac events (MACE) than AA genotype carriers [22,23]. Moreover, the G allele appears to affect the expression levels of the gene, reducing the mRNA amounts [23]. Thus, similarly to the findings of Saito and colleagues [19], it might be that low levels of cPLA2α reduce the risk of cardiovascular diseases. In patients with nephrosclerosis, a pathology increasing the risk of CVDs, an intronic variant in *PLA2G4A* (rs72709847) has been associated with the presence of atherosclerotic plaques, and two other variants (rs932476 and rs6683619) have been associated with decreased survival without a cardiovascular event [24]. There are also rare and non-synonymous mutations in this gene related to platelet dysfunction and intestinal ulcers, which are caused by a consequent minor synthesis of thromboxane A2 [25]. Indeed, it seems that cPLA2α plays a central role in hemostasis [26].

#### 3.1.2. Cytosolic Ca^2+^-Dependent PLA2ζ and Ca^2+^-Independent iPLA2γ

In contrast, there is another poorly characterized phospholipase A2 whose normal activity seems to have a beneficial role. Cytosolic Ca^2+^-dependent PLA2ζ (*PLA2G4F*) is expressed in myocardial mitochondria, and it appears that AA hydrolyzed by this enzyme is preferentially associated with CYP450 epoxygenases to produce EETs, determining a cardioprotective role in non-failing human myocardium. In contrast, a remodelling of phospholipases happens during heart failure, determining Ca^2+^-independent iPLA2γ activation, which channels the AA into the synthesis of cardiotoxic HETEs and promotes the opening of the mitochondrial permeability transition pore (mPTP) [27]. This opening in turn induces the release of reactive oxygen species (ROS), pro-apoptotic factors, and the subsequent death of cardiomyocytes, increasing the reperfusion injury [28]. It has previously been shown that loss of function in iPLA2γ^−/−^ knockout mice reduces the opening of mPTP [29] and the size of the infarcted area in the heart [30]. As cPLA2ζ, iPLA2γ is located on mitochondrial membranes, and it is encoded by the *PNPLA8* gene [31].

### 3.2. Secretory PLA2s

#### 3.2.1. Secretory PLA2-IIA

Among secretory PLA2s, sPLA2-IIA, encoded by the *PLA2G2A* gene and also referred to as an inflammatory PLA2, has been associated with cardiovascular diseases. This enzyme hydrolyzes both arachidonic acid and lipoproteins, is involved in host defense, and is also up-regulated during inflammation [15]. It is induced by pro-inflammatory cytokines, bacterial lipopolysaccharides [32], and mitochondria released by platelets, determining the production of lipid mediators and thus amplifying inflammation [33,34]. High levels of this enzyme have been implicated in different cardiovascular diseases, such as atherosclerosis and coronary artery disease. It is overexpressed in atherosclerotic plaques, specifically in macrophages which infiltrate and cause intima thickening, and enhances atherosclerosis development through following prostaglandin synthesis and even lipid deposition [20,35,36,37,38]. Similarly, this enzyme is up-regulated in epicardial adipose tissue and has greater activity in patients affected by coronary artery disease [39,40].

In the JUPITER trial, in which rosuvastatin or a placebo were administered to participants with systemic inflammation to evaluate the association of sPLA2-IIA with its plasma levels and cardiovascular disease incidence, it was discovered that high sPLA2-IIA levels are associated with a higher risk of CVD. Moreover, three polymorphisms in *PLA2G2A* (rs11573156, rs2307246, and rs4744) have been related to sPLA2-IIA plasma levels, and the intronic variant rs11573156 has a strong effect on its levels, where GG genotype carriers have higher levels of the enzyme [41]. This study confirms previous findings that the C allele of rs11573156 is associated with lower levels and reduced activity of sPLA2-IIA, while greater levels are related to the G allele [42,43].

#### 3.2.2. Secretory PLA2-V

Another secretory PLA2 highly associated with cardiovascular disease is sPLA2-V, encoded by *PLA2G5* [15]. Similar to sPLA2-IIA, high levels of sPLA2-V are implicated in atherosclerosis development [38]. This enzyme is not expressed normally in the heart, but its expression rapidly increases in myocardium after the onset of myocardial infarction, followed by a great production of COX-2 [44]. In mice, it is upregulated after myocardial infarction, increasing the reperfusion injury, while in knockout sPLA2-V^−/−^ mice there is a reduction of myocardial apoptosis [45].

The intronic variant rs525380 influences the expression of *PLA2G5* and the A allele is related to gene upregulation; however, it does not seem to be casually related to the pathogenesis of cardiovascular diseases [46]. In contrast, the intronic variant rs11573191 has been related to a higher risk of early coronary artery disease and hypertension in the Mexican Mestizo population [47].

## 4. Cyclooxygenases: The Key Point of Prostanoids Synthesis

After arachidonic acid cleavage, cyclooxygenases and other enzymes give rise to prostanoids, a class of heterogeneous bioactive eicosanoids with pro-inflammatory, anti-inflammatory, and pro-thrombotic roles [6,7,8,9,10]. In the first step of prostanoid synthesis, released AA is converted by cyclooxygenases into the highly unstable prostaglandin PGG2, which is rapidly reduced to PGH2, the substrate for prostanoid synthesis. Prostanoids comprise prostaglandins, prostacyclin, and thromboxane A2 [48]. Owing to their functions, they have been widely associated with different types of CVDs, and the enzymes involved in their synthesis are the target of anti-inflammatory drugs [7,49].

Cyclooxygenases COX-1 and COX-2, which are also known as prostaglandin endoperoxide synthases and are encoded by *PTGS1* and *PTGS2* genes, respectively, are the rate-limiting enzymes for the synthesis of prostanoids. Generally, COX-1 is constitutively expressed in several tissues, while COX-2 is inducible by pro-inflammatory cytokines (for example IL-1β and TNF-α produced during pathogen invasion) and is up-regulated in inflammatory conditions, such as atherosclerosis. Nonetheless, even COX-1 is inducible by pro-inflammatory mediators in several cell types [50,51]. The two cyclooxygenase isoforms differ in the profile of prostanoids synthesized by them. Indeed, COX-1 activity channels PGH2 towards the production of prostacyclin (PGI2), thromboxane A2 (TxA2), prostaglandin D2 (PGD2), and 12-hydroxyheptadecatrienoic acid (12-HETE); on the contrary, COX-2 promotes the synthesis of prostacyclin and prostaglandin E2 (PGE2) [52]. Overall, their activity is implicated in inflammation enhancement or resolution and in thrombosis; COX-2 especially promotes inflammation, while COX-1 mostly induces thrombosis [52].

Both cyclooxygenases are inhibited by non-steroidal anti-inflammatory drugs (NSAIDs), among which aspirin has been shown to impair thrombosis and platelet function by blocking thromboxane A2 formation [53]. On one hand, selective pharmacological inhibition of COX2 reduces inflammation, but on the other hand, it has been demonstrated that this inhibition increases vascular thrombosis in mice. This is determined by a consequent reduction of prostacyclin, which is a potent vasodilator and antithrombotic [54,55,56].

Several polymorphisms in COX genes have been identified as risk or protective factors against many pathologies, not only cardiovascular diseases. A common variant in the *PTGS2* promoter, rs20417 (-765G > C), has been extensively studied because it influences gene transcription, prostanoids synthesis, and susceptibility to CVD. Two meta-analyses re-analyzed the association studies on this variant and found that the CC genotype reduces the risk of coronary artery disease (CAD) [57,58]. However, there are opposing data regarding this polymorphism. The derived C allele, in contrast to the G allele, is associated with lower promoter activity by altering the binding of transcription factor Sp1 [59] and with lower plasma levels of inflammation markers interleukin-6 (IL-6) [60], C-reactive protein (CRP), and pro-thrombotic von Willebrand factor [61]. Therefore, it protects against myocardial infarction, atherosclerotic ischemic stroke, and cerebrovascular ischemic disease in general [62,63], as well as reducing carotid intima-media thickness [61]. A prospective study involving about 49,000 individuals from different trials has shown that carriers of the C allele present a reduced risk of major cardiovascular outcomes, vascular death, myocardial infarction, and stroke [64]. Moreover, a recent study on the Korean population has shown an association between this polymorphism, mostly the GG genotype, and CAD [65]. The same correlation was previously discovered in the Chinese Uyghur population, indicating a protective role of the C allele [66]. Unlike the findings in Caucasian and Asian individuals [61,62,63,64,65,66], the Atherosclerosis Risk in Community (ARIC) Study has shown that the C allele would increase the risk of stroke in African-Americans [67,68]. Similarly, the Helsinki Sudden Death Study has found that individuals carrying the CC genotype have larger atherosclerotic and complicated lesions in their coronary arteries than individuals with the GG genotype [69]. Finally, two studies did not find any association between rs20417 and CVDs [70,71]. Therefore, these differences may be due to the small sample size of some of these studies or might indicate a different impact of geographical ancestry on this variant. It is also interesting to note that the derived allele would strengthen the effect of aspirin in some studies [64,72], but the same was not found in another study [67]. In contrast, it has been related to aspirin resistance in Indian patients affected by stroke [73].

Regarding *PTGS1*, it presents several polymorphisms in the promoter region, which are in strong linkage disequilibrium in Europeans and Africans, and several exonic substitutions, influencing gene expression and enzymatic activity. Of note, some of the identified missense variants lower the enzymatic activity, likely reducing the risk of developing cardiovascular diseases, too [74]. The polymorphism -1006G > A, located in the 5′-UTR of the gene, has been associated with risk of ischemic cardiovascular disease and stroke (the A allele) in Caucasians involved in the ARIC study [67]. Conversely, the minor G allele of rs10306114 in the *PTGS1* promoter region appears to decrease the incidence of myocardial infarction in Caucasian patients affected by coronary artery disease [75]. In Caucasians, this SNP is in linkage with the exonic variant rs3842787 [74], which determines the aminoacidic substitution Pro17Leu (C > T substitution). Both rs10306114 and rs3842787 have been detected as risk factors for bleeding complications due to elective coronary angiography, more specifically the minor G and T alleles, respectively [76]. The missense variant rs3842787 also plays a role in aspirin response; indeed, the CC genotype appeared to reduce the response to aspirin [77], while the derived T allele is more sensitive to inhibition by another NSAID drug [74]. Still relating to CVD risk, the CC genotype of rs1330344 (2KB upstream variant) might increase the risk of adverse events in Chinese patients with previous ischemic stroke treated with aspirin [78]. It is also strongly associated with higher susceptibility to Kawasaki disease in Chinese children [79]. Moreover, the AA genotype of the same SNP seems to diminish the risk of ischemic stroke [80]. Similar to *PTGS2*, there are differences among distinct geographical ancestries.

Therefore, genetic variants in COX genes can lower or increase the risk of CVD pathogenesis by directly influencing the following synthesis of prostaglandins, prostacyclin, and thromboxane A2.

## 5. Prostanoids

There are four types of prostanoids resulting from isomerization of PGH2 by prostaglandin synthases, namely prostaglandin D2, prostaglandin F2α, prostaglandin E2, prostacyclin, and thromboxane A2. All prostanoids are autacoids and exert their action through the interaction with specific G protein-coupled receptors on cellular membranes, determining their pro-inflammatory or anti-inflammatory effect. Each receptor elicits different cellular responses involving distinct molecular mediators, as displayed in Table 1. Several studies have reported that prostaglandins are involved not only in acute but also in chronic inflammation. Overall, prostaglandins are modulators of inflammation [7,81,82]. In the following paragraphs, we will focus on each prostanoid.

### 5.1. Prostaglandin D2 (PGD2)

Prostaglandin D2 is synthesized in several tissues, both in the central nervous system and in peripheral tissues; among the latter, it is expressed in cells involved in immune and inflammatory responses, endothelial cells, and cardiomyocytes [83,84,85,86]. It is produced by two different PGD synthases, the lipocalin-type prostaglandin D synthase (L-PGDS) and the hematopoietic prostaglandin D synthase (H-PGDS). It exerts its action by interacting with two distinct receptors: PGD2 receptor subtype 1 (DP1) and a chemoattractant receptor-homologous molecule expressed on Th2 lymphocytes (CRTH2) [86]. PGD2 is quickly converted into bioactive metabolites in a reaction partially dependent on serum albumin [87].

**Figure 2:**
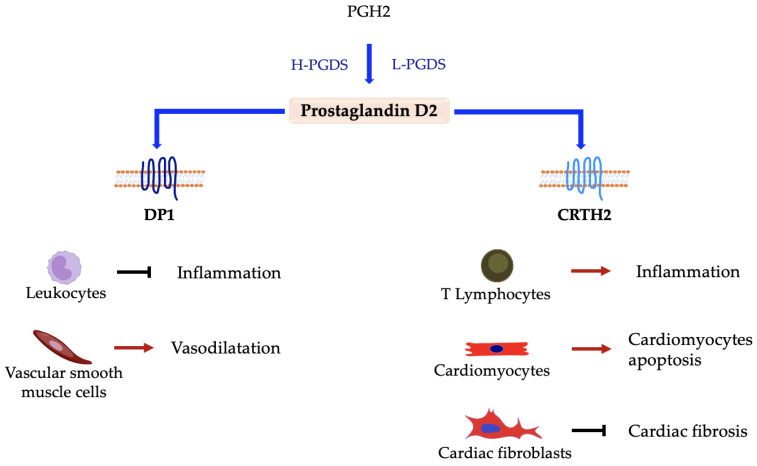
Responses elicited in different cell types by interaction between prostaglandin D2 and distinct receptors. → denotes induction; ⊣ denotes inhibition.

PGD2 plays both pro-inflammatory and anti-inflammatory roles, depending on tissue expression and even receptor interaction (Figure 2). On one hand, it promotes allergic inflammation of the airways, while on the other hand, it is involved in the resolution of non-allergic inflammation. It seems to play a role in cardiovascular homeostasis and to play a beneficial role in several cardiovascular diseases [86]. In the context of allergic responses, for instance, it is highly expressed in IgE-activated mast cells, exerting a bronchoconstrictor effect and promoting pulmonary hypertension through the interaction with DP1 receptors located on bronchial epithelial cells. Moreover, it induces inflammation and cytokine release by interacting with CRTH2 on type 2 T helper cells [83,88,89,90,91,92]. It is also interesting to note that the number of CRTH2^+^ cells is correlated to PGD2 levels [92]. Both PGD2 and CRTH2 receptors are expressed in cardiomyocytes, and the PGD2/CRTH2 axis plays a detrimental role in the setting of myocardial ischemia and heart failure. Specifically, the hypoxic condition determines the upregulation of the PGD2/CRTH2 axis, which in turn promotes the apoptosis of cardiomyocytes through caspase activation [31]. The CRTH2 receptor is even expressed in cardiac fibroblasts [93], and the interaction with PGD2 determines the trafficking and the anchoring of CRTH2 to the endoplasmic reticulum (ER) membrane [94]. However, in contrast to its injurious role on cardiomyocytes [93], the CRTH2 localized on the ER membrane inhibits collagen synthesis, while the deletion of this receptor increases cardiac fibrosis and dysfunction [94]. The PGD2/CRTH2 axis thus plays a detrimental or beneficial role in the heart depending on cell type expression.

**Figure 3:**
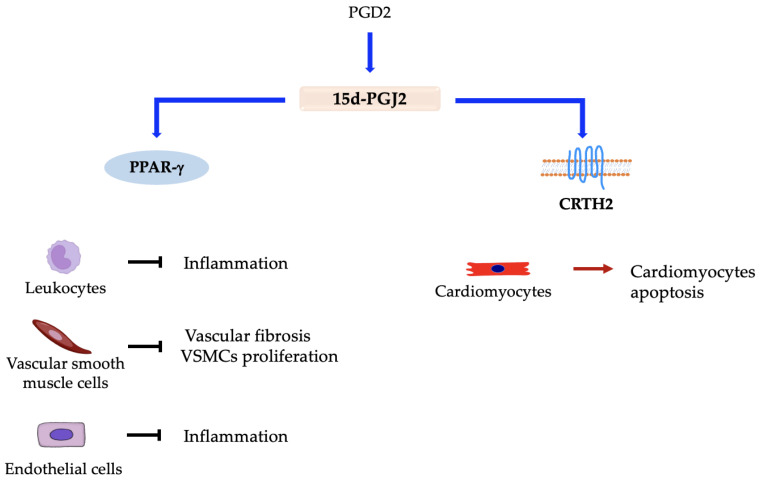
Responses elicited in different cell types by interaction between prostaglandin 15d-PGJ2 and distinct receptors. → denotes induction; ⊣ denotes inhibition.

On the other hand, the anti-inflammatory role of PGD2 is mainly mediated by its degradation product, 15-Deoxy-∆-^12,14^-prostaglandin J2 (15d-PGJ2), which controls the balancing of cytokine synthesis and leads to inflammation resolution (Figure 3) [95,96]. 15d-PGJ2 anti-inflammatory role is mediated through interfacing with peroxisome proliferator-activated receptor *γ* (PPAR-*γ*). This bond modulates T-cell activation and blocks macrophage migration. Moreover, when interacting with PPAR-*γ* on endothelial cells, 15d-PGJ2 inhibits the nuclear factor kappa-light-chain-enhancer of activated B cells (NF-κB) and the secretion of pro-inflammatory cytokines [97]. Through PPAR-*γ* activation, 15d-PGJ2 reduce the progression of atherosclerosis in vascular smooth muscle cells (VSMCs) and vascular fibrosis by inhibiting the activity of transforming growth factor *β* (TGF-*β*) and collagen deposition [98]. It is also able to inhibit the proliferation of VSMCs, whose spread is indicative of atherosclerosis development [99]. However, it has been found that 15d-PGJ2 is able to interact with CRTH2 receptor on cardiomyocytes, promoting expression of TNF-*α* and subsequent cardiomyocyte apoptosis [100]. In addition to 15d-PGJ2, even PGD2 plays a role in the resolution of inflammation. During myocardial infarction, when PGD2 interacts with the DP1 receptor on macrophages, it determines the polarization of these cells and improves the resolution of inflammation in the heart [101].

#### Prostaglandin D Synthases and Receptors

PGD2 is synthesized by two different PGDS enzymes, which differ in their tissue expression. In fact, L-PGDS is expressed in many cell types, such as cardiomyocytes and endothelial cells [48,102,103], while H-PGDS is mainly expressed in antigen-presenting cells, such as macrophages and mast cells [48,84,104]. The L-PGDS and H-PGDS are encoded by two different genes, which are *PTGDS* and *HPGDS*, respectively.

It has been shown that deletion of L-PGDS is associated with an increase in hypertension and thrombosis in mice [105] and a greater and more rapid development of atherosclerosis [106]. In line with these results, the L-PGDS-derived PGD2 determines cardiac protection against myocardial ischemia and reperfusion injury [107]. This effect is mainly determined by the 15d-PGJ2/PPAR-γ axis [107], but also through interaction of PGD2 with the PGF2α receptor [108]. In contrast, as mentioned above, activation of the CRTH2 receptor by 15d-PGJ2 induces the apoptosis of cardiomyocytes [100]. Similarly, H-PGDS deficiency impairs the resolution of acute inflammation, resulting in the accumulation of macrophages and lymphocytes [96].

Some *PTGDS* polymorphisms, which determine aminoacidic substitutions, enhance or reduce the catalytic activity of L-PGDS [109], promoting the disruption of PGD2 synthesis and likely the development of pathologic states.

Similar to PGD2 synthases, the PGD2 receptors are expressed in different cell types. DP1 is mostly expressed in leukocytes and the vascular system, while CRTH2 is highly expressed in immune cells [86], even in the heart [93,94]. The PGD2/DP1 axis is involved in the regulation of blood pressure, promoting vasodilatation of pulmonary arteries [110]. Patients affected by idiopathic pulmonary arterial hypertension have lower expression of DP1, and its deletion exacerbates this pathology by increasing vascular smooth muscle cell hypertrophy and proliferation, possibly leading to heart failure [111]. Moreover, the activation of the DP1 receptor counteracts the hypertension caused by angiotensin-II, blocking the transition of VSMCs into myofibroblast and the thickening of vascular media [112]. It has been shown that niacin drug therapy induces the release of PGD2 in macrophages, localized in the heart, and improves wound healing and cardiac function after myocardial infarction. In particular, the interaction of PGD2 with the DP1 receptor on macrophages determines the polarization of these cells into anti-inflammatory macrophages (M2), promoting the resolution of inflammation [113].

### 5.2. Prostaglandin F2α (PGF2α)

The prostaglandin PGF2α is produced using PGH2 or PGD2 as substrate by prostaglandin F synthase (PGFS, also known as aldo-keto reductase (AKR) 1C3) and PGE2 by prostaglandin E2 9-ketoreductase (also known as carbonyl reductase 1) [114]. It interacts with the prostaglandin F (FP) receptor. PGF2α is expressed in many tissues, among which are heart and vascular tissues, but it plays a main role in the female reproductive system, in which it is involved in ovulation, luteolysis, uterine contraction, and the initiation of parturition [115,116].

**Figure 4:**
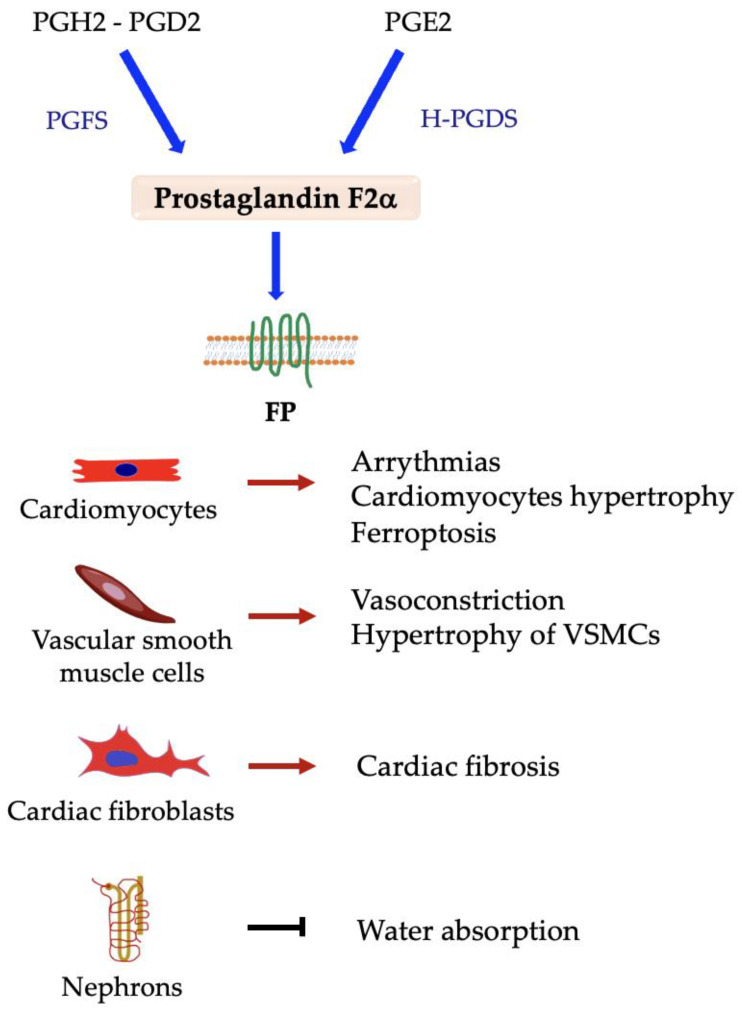
Responses elicited in different cell types by interaction between prostaglandin F2*α* and FP receptor. → denotes induction; ⊣ denotes inhibition.

Several studies have shown that PGF2α is associated with cardiovascular diseases. It is implicated in acute and chronic inflammation that may lead to the development of different CVDs (Figure 4), such as atherosclerosis and ischemia-reperfusion injury [117,118]. It has been demonstrated that both PGF2α and TxA2, whose synthesis is stimulated by cytokines in inflammatory conditions, are implicated in the development of arrythmias. They increase the beating rate of the right atrium of mice through the interaction with respective receptors on cardiomyocytes, then induce inflammatory tachycardia; in FP knockout mice, this increase was not observed [119]. It has also been reported that PGF2α is highly produced during myocardium inflammation and reduces the contractility of cardiomyocytes in the right ventricle of the rat heart, likely contributing to heart failure [120]. In the cardiovascular system, PGF2α leads to vasoconstriction of VSMCs and hypertrophy of both VSMCs and cardiac myocytes [121,122,123], since it induces the expression of NOX1 and the following O^2−^ production [124] when it interacts with FP receptors on these cell types. In cardiac fibroblasts, it promotes collagen deposition in a dose-dependent way, contributing to myocardial fibrosis development and undermining heart function. Instead, FP receptor inhibition reduces the fibrosis and protects from diabetic cardiomyopathy [125,126].

It plays a vasoactive and hypertensive role through its interaction with the prostaglandin F receptor in VSMCs and in the kidney. It seems that PGF2α inhibits water and salt absorption in the kidney [127], and its renal expression is increased by salt [128]. In wild-type mice, PGF2α elevates the blood pressure by indirectly inducing the secretion of renin and accelerates the pathogenesis of atherosclerosis in the presence of a high-fat diet. In contrast, the deletion of the FP receptor determines blood pressure reduction and a slower development of the same pathology, as reported in [129]. FP receptor activity seems to increase the aging of VSMCs and their dysfunction, which is delayed by FP receptor deletion [130]. Overall, it is possible to state that PGF2α/FP axis plays a critical role in cardiovascular dysfunction, being involved in the development of atherosclerosis, hypertension, and cardiovascular fibrosis.

#### Prostaglandin F Synthases and FP Receptor

As mentioned, PGF2α is synthesized by two different enzymes [48]. The aldo-keto reductase 1C3 is encoded by *AKR1C3* and is expressed in several tissues. It metabolizes other molecules and is involved in cancer development and drug metabolism and resistance, too [131]. A meta-analysis has displayed that this gene is markedly downregulated in patients with acute myocardial infarction (AMI) and seems to be involved in ferroptosis of cardiomyocytes [132], a form of non-apoptotic cell death induced by iron-dependent accumulation of toxic lipid ROS in cells [133]. However, another meta-analysis has shown that *AKR1C3* is upregulated in patients affected by AMI [134]. Maybe, the difference is due to different analyses performed and a different number of datasets employed. Overexpression of AKR1C3, stimulated by estrogen receptor β (ESR2) activity, appears to exert vasoconstriction of the fetoplacental endothelium [135]. It is also implicated in preeclampsia [136], a pregnancy disorder characterized by hypertension, and the G allele of the rs10508293 variant is associated with a lower risk of preeclampsia, while the A allele is related to high blood pressure in Han Chinese women [137]. Therefore, population ancestry might have a different influence on this polymorphism.

The carbonyl reductase 1 is encoded by the *CBR1* gene. In addition to PGE2, it has a broad specificity of substrate and is expressed in many tissues. CBR1 is involved in the metabolism of anticancer drugs and produces cardiotoxic compounds from the metabolization of these drugs; in contrast, its inhibition exerts a cardioprotective role [138,139]. Furthermore, an aminoacidic substitution, Val88Ile, reduces the kinetics of anthracyclines and PGE2 metabolization [140]. A recent study has demonstrated that CBR1 is likely involved in cardiac ischemia/reperfusion (I/R) injury since its downregulation might reduce the oxidative stress induced by ROS produced during I/R injury [141].

The FP receptor is encoded by the *PTGFR* gene. As stated, the FP receptor mediates the detrimental role of PGF2α in the cardiovascular system, while its deletion often has a beneficial role. Some polymorphisms in *PTGFR* have already been reported to be associated with the lanoprost response and with an increase in intraocular pressure in patients affected by glaucoma [142]. It has been discovered that the 3′-UTR variant rs12731181 alters the binding affinity of miR-590-3p to PTGFR mRNA, promoting receptor overexpression and increasing the risk of hypertension in a Chinese population [143].

### 5.3. Prostaglandin E2 (PGE2)

**Figure 5:**
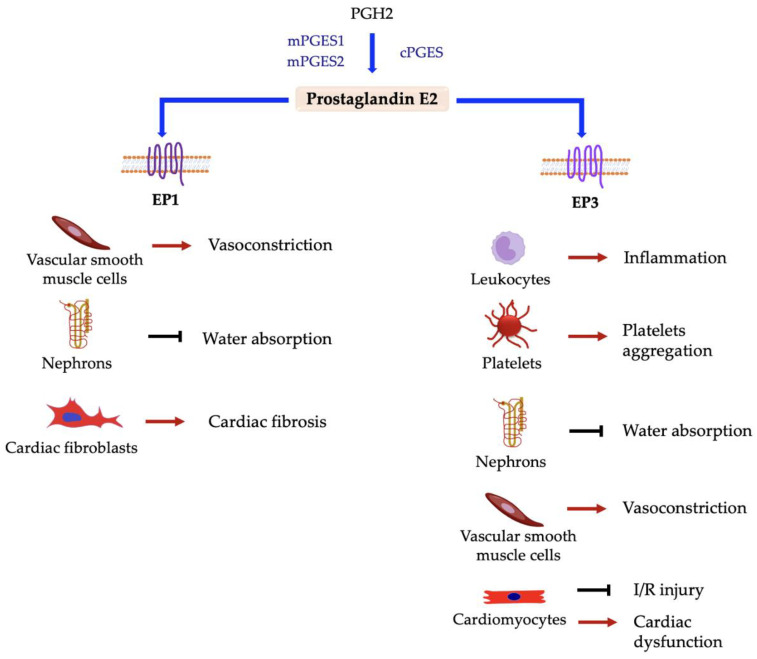
Responses elicited in different cell types by interaction between prostaglandin E2 and EP1 and EP3 receptors. → denotes induction; ⊣ denotes inhibition.

**Figure 6:**
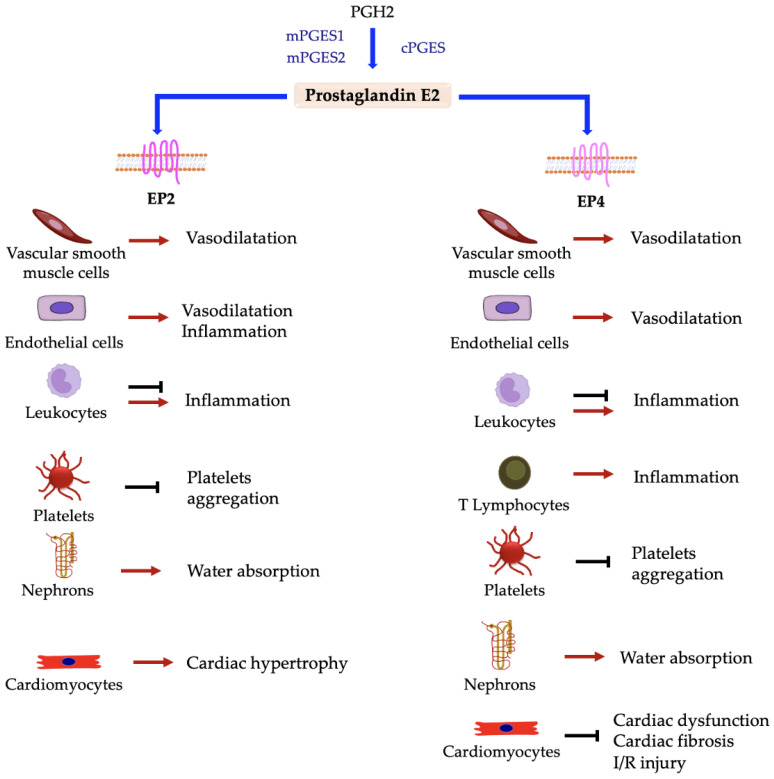
Responses elicited in different cell types by interaction between prostaglandin E2 and EP2 and EP4 receptors. → denotes induction; ⊣ denotes inhibition.

Prostaglandin E2 is maybe the best characterized prostaglandin. It is mainly known as an inflammation mediator and exerts its action via the interaction with four different receptors, E-type prostanoid receptors 1–4 (EP1-4), that are widely expressed in platelets and VSMCs [10,144,145]. PGE2 is synthesized in many cell types, including endothelial cells, platelets, macrophages, and fibroblasts, and is rapidly inactivated into 15-keto-PGE2 by 15-hydroxyprostaglandin dehydrogenase (15-PGDH or prostaglandin dehydrogenase 1) [146]. It is involved in many processes and in the pathogenesis of several diseases. Its synthesis is induced by both COX-1 and COX-2 activity, but in pathological conditions there is a correlation between COX-2 and PGE2 synthesis [52]. Overall, PGE2 have different effects on various diseases depending on receptor interaction, with effects can ameliorate or worsen the pathological condition [147]. Figure 5 and Figure 6 show the effect of PGE2 on different cell types based on receptor interaction.

#### 5.3.1. PGE2 and Inflammation

Many studies have displayed that it is greatly expressed during inflammation, promoting the synthesis and secretion of cytokines and giving rise to proinflammatory or anti-inflammatory responses. The different responses are mainly due to the interaction with distinct receptors and the timing of exposition [10,144,145].

During acute inflammation, caused by tissue damage or pathogen invasion, COX-2 induction by pro-inflammatory stimuli raises PGE2 expression [52], which mediates acute inflammatory responses (red flares, heat, and swelling reactions). Specifically, PGE2 triggers vasodilatation, blood flow, and an increase in vascular permeability by interacting with EP2, EP3, and EP4 receptors in vascular smooth muscle cells [144,148,149]. The interaction between PGE2 and EP3 on mast cells elicits the release of pro-inflammatory mediators, such as histamine and also interleukin-6 (IL-6). These mediators augment vascular permeability and promote consequent leukocyte recruitment and edema. Thus, the PGE2/EP3 axis is implicated in acute inflammation [150]. On the other side, EP2/EP4 stimulation of vascular smooth muscle cells and endothelial cells determines vasodilatation and the subsequent increase in blood flow [151].

Through EP2 and EP4 receptors, PGE2 regulates pro-inflammatory interleukin-1β secretion induced by inflammasome activation [152,153,154,155,156,157], a process that, if not properly regulated, leads to cell death and possibly the development of autoimmune diseases [158]. However, there are contrasting data points in this regard. Some studies have shown that PGE2, when interacting with the EP4 receptor, inhibits the NLRP3 inflammasome and IL-1β secretion on monocytes, exerting an anti-inflammatory role [152,153,154]. In contrast, other studies have discovered that this prostaglandin stimulates IL-1β synthesis and secretion [155,156,157] when it interacts not only with EP3 but even with EP2 and EP4 receptors. These differences are likely due to the different timing of exposition to PGE2, in addition to receptor binding.

PGE2 is also involved in chronic inflammation, which is characterized by the persistence of an inflammatory state due to the continuous production of pro-inflammatory cytokines, low efficiency of inflammation resolution, and protracted leukocyte recruitment. All of these events can result in abnormal tissue remodelling and damage, eventually leading to disease. Among chronic inflammatory conditions, PGE2 plays a role in cancer, diabetes mellitus, and cardiovascular diseases [8,159,160]. It has been proposed that this promotes chronic inflammation in a positive feedback loop. On one hand, the PGE2/EP2 axis activates in endothelial cells the NF-κB factor, which in turn induces the cytokine synthesis and the indirectly triggered activation of COX-2 triggered by the same cytokines [161]. On the other hand, the PGE2/EP4 axis enhances differentiation of T cells into Th1 (type 1 T helper) cells and Th17 expansion, due to the release of IL-12 and IL-23, increasing the inflammation [162].

#### 5.3.2. PGE2 and Atherosclerosis

Among cardiovascular diseases, PGE2 has been widely associated with atherosclerosis [8,9,160], a pathology characterized by vascular chronic inflammation that leads to vascular obstruction, macrophage infiltration, ischemic syndromes, and coronary artery disease development [163]. Nevertheless, there are contrasting data in this regard.

During atherosclerosis, PGE2 is overexpressed in macrophages localized in atherosclerotic plaques, thanks to COX-2 and PGE synthase upregulation in the same cells. PGE2 in turn increases inflammation through cytokine secretion and macrophage activation. In addition to cytokines, even matrix metalloproteinases are secreted by activated macrophages and degrade the constituents of atherosclerotic plaque, contributing to plaque instability and rupture [164]. This PGE2 pro-inflammatory activity on atherosclerotic plaques is mediated by EP4 receptor overexpression in macrophages [165].

At the same time, it has been demonstrated that PGE2 interacting with EP4 plays an anti-inflammatory role, since it reduces the expression of several pro-inflammatory mediators, such as IL-8, in human activated macrophages. Thus, it appears that it diminishes the propagation of inflammation [166]. It has also been shown that this anti-inflammatory role is triggered by PGE2-induced production of anti-inflammatory IL-10 and activation of EP4-receptor associated protein (EPRAP), which inhibits pro-inflammatory NF-κB1 in macrophages [167]. PGE2/EP2-EP4 axis promotes the polarization of pro-inflammatory M1 macrophages into anti-inflammatory M2 macrophages by activating the CREB (cAMP response element-binding protein) signaling pathway, too [168].

The different roles of the EP4 receptor in stimulating pro- or anti-inflammatory signals likely depend on atherosclerosis phases [147]. Furthermore, the interaction between PGE2 produced in atherosclerotic plaques and EP3 receptor on platelets promotes platelet aggregation, aggravating atherothrombosis [169,170]. In contrast, EP3 deletion limits atherothrombosis [171], as well as the activation of EP2 and EP4 receptors on platelets, which inhibit their aggregation [172].

#### 5.3.3. PGE2 and Blood Pressure

PGE2 was associated with other CVDs. PGE2 has been linked to abdominal arterial aneurysm rupture [173]. In the kidney, it is implicated in water and salt reabsorption regulation, thus controlling blood pressure, and it exerts both vasodilatory and vasoconstrictory action depending on receptor interaction. When it interacts with EP1 and EP3, it induces vasoconstriction, while it promotes vasodilatation when binding to EP2 and EP4 receptors. It is also involved in angiotensin-II-induced vasoconstriction [147,174].

PGE2-EP1 interaction in the collecting duct of nephrons attenuates water reabsorption and blocks sodium transport [175,176]. EP1 was associated with hypertension, and its disruption lessens the mean arterial pressure and the risk of end-organ damage [177]. Furthermore, activation of EP1 in vascular vessels exerts vasoconstriction by directly promoting contraction of vascular smooth muscle cells [178]. EP3 receptors have the same effect as EP1 receptors in the kidney, since they inhibit the reabsorption of fluids [179] and the relaxation of vascular smooth muscle cells in vessels. As corroboration, EP3 deletion lowers the blood pressure [180].

In contrast, PGE2-EP2 and PGE2-EP4 interactions in the nephrons collecting duct promote water reabsorption by increasing aquaporin trafficking and consequent vasodilatation [181,182]. Moreover, EP4 expressed in vessels determines vasodilatation [183], and its deletion on VSMCs in angiotensin-II-infused mice is associated with hypertension, vascular inflammation, and the risk of developing aortic dissection [184]. EP2 promotes renal excretion of sodium in the presence of high-salt diet, lowering blood pressure [185]. Thus, PGE2/EP1-EP3 axes induce vasoconstriction and hypertension, while PGE2/EP2-EP4 axes induce vasodilatation. PGE2 and EP receptors thus play an important role in the pathogenesis of hypertension and possibly of myocardial ischemia and heart failure [186].

#### 5.3.4. PGE2 and Cardiac Health

Regarding myocardial infarction, PGE2 plays both a protective role and a detrimental role depending on receptor involvement and cell type. In addition, the same receptors are able to elicit different cellular responses, which determine distinct effects.

During acute myocardial ischemia, due to obstruction of coronary arteries, an inflammatory response occurs, which gives rise to prostaglandin production. PGE2 inhibits contraction of cardiac myocytes induced by β-adrenergic stimulation when it interacts with the EP4 receptor [187] and it protects against contractile dysfunction associated with heart failure [188]. The PGE2/EP4 axis also blocks the synthesis and secretion of TGF-β1 factor in cardiomyocytes, preserving the heart from cardiomyocyte hypertrophy and cardiac fibrosis because TGF-β1 increases the synthesis of collagen in fibroblasts [189]. It has also been found that EP4 overexpression improves cardiac function after myocardial infarction via the inhibition of pro-inflammatory cytokine secretion and the deposition of collagen. This results in an improvement of the ejection fraction and a reduction of macrophage infiltration [190]. The interaction with EP4 stimulates hypertrophy of cardiomyocytes during myocardial infarction, too [191]. In contrast, EP4 deletion only in cardiomyocytes eases cardiac hypertrophy and remodelling [192], while global cardiac EP4 deletion increases the infarct size [193,194]. This indicates a dual role of the PGE2/EP4 axis on myocardial ischemia, but it would appear that it has a prevailing beneficial role.

More recent studies on the EP3 receptor state that it plays a detrimental role in heart failure, but there are conflicting results regarding EP3 action on cardiomyocytes. Some studies have shown that it protects the infarcted heart from reperfusion injury and reduces the infarct size [195,196,197]. In addition, EP3 overexpression reduces the cardiomyocytes’ contraction [198,199] and augments the hypertrophy of these cells [200]. In contrast, it has been discovered that the PGE2/EP3 axis activates MEF2 transcription factors in cardiomyocytes after cardiac inflammation, promoting adverse cardiac remodelling and, ultimately, heart failure [201]. Furthermore, EP3 overexpression, as it happens after heart failure, is associated with a lower ejection fraction, increased left ventricle dimension, impaired cardiomyocyte contractility and relaxation, greater collagen deposition, and macrophage infiltration. Thus, it seems to exert a detrimental role on cardiac function [202], but, as EP4, it might determine different responses.

The binding of EP1 receptors to cardiac fibroblasts seems to increase their proliferation and collagen deposition, worsening cardiac recovery after ischemia [203]. Instead, EP2 receptors appear to induce cardiac hypertrophy after the interaction with PGE2 in cardiomyocytes [204].

#### 5.3.5. PGE2 Synthases

PGE2 is synthesized from PGH2 by three different prostaglandin E synthases: cytosolic PGE2 synthase (cPGES), microsomal PGE2 synthase 1 (mPGES-1), and microsomal PGE2 synthase 2 (mPGES-2) [48]. cPGES and mPGES2 are constitutive enzymes expressed in several cell types, whereas mPGES1 is mainly induced by pro-inflammatory IL-1β [205,206].

The microsomal PGE synthase 1 is encoded by the *PTGES* gene. Its expression in macrophages increases as a consequence of pro-inflammatory stimuli and is coupled to COX-2 upregulation [205,207,208]. Several studies have shown that mPGES-1 is involved in cardiovascular diseases [209]. It has been found that COX-2 and mPGES-1 are overexpressed in peripheral blood mononuclear cells and atherosclerotic plaques of patients with carotid stenosis, fostering inflammation [210]. Instead, its deletion slows down atherosclerosis development since PGH2 is channeled into PGI2 synthesis [211,212]. mPGES-1 is implicated in vascular dysfunction in hypertensive patients because it participates in vasoconstriction and increases vascular oxidative stress through ROS production. In contrast, its deletion in vascular smooth muscle cells prevents these pathogenic events [213].

On the other hand, it has been shown that it is protective against cardiac ischemia and reperfusion injury. Deletion of mPGES-1 during acute cardiac ischemia increases the infarct size and promotes myocardial ischemia/reperfusion injury; moreover, the mPGES1/PGE2/EP4 axis limits the interaction between leukocytes and endothelial cells of coronary arteries in the context of I/R injury, protecting the heart and preserving microcirculation during the reperfusion phase [194]. As said, *PTGES* expression is induced by cytokines and other factors. It has also been demonstrated that its expression is inhibited by PPAR-γ activated by 15d-PGJ2 [214,215]. Actually, polymorphisms in this gene have not yet been identified as risk factors for cardiovascular diseases.

mPGES-2 is encoded by the *PTGES2* gene, and its expression is linked to both COX-1 and COX-2 activity [216]. However, a study has demonstrated that mPGES-2 does not seem to contribute to PGE2 production in vivo, but its deletion in the heart influences the expression of other genes [217]. There are no variants in the *PTGES2* gene associated with CVDs.

cPGES is encoded by the *PTGES3* gene, which is permanently expressed and unaffected by inflammatory stimuli. Its expression is functionally coupled to that of COX-1, playing a role in the constitutive synthesis of PGE2 [218]. It does not appear to be involved in the pathogenesis of cardiovascular diseases; however, some genetic variants in the *PTGES3* gene have been associated with cardiovascular functions through genome-wide association studies. In particular, it was related to QRS duration (rs2958145 [219]), atrial fibrillation (rs2860482 [220], rs7978685 [221,222]), diastolic blood pressure (rs7137749 [223]), mean platelet volume (rs2950390 [224], rs2958154 [225]), and count (rs2958154 [225]). Nevertheless, they need to be further investigated.

#### 5.3.6. Prostaglandin Dehydrogenase 1

As yet mentioned, PGE2 is degraded by the 15-PGDH enzyme (encoded by the *HPGD* gene), and its dysfunction promotes the pathogenesis of several diseases. It plays a critical role in the regulation of inflammation, and a reduction in its expression is associated with the development of this pathological condition [226]. 15-PGDH activity promotes cardiac fibrosis, even in the infarcted heart, owing to PGE2 degradation; it has been shown that its deletion promotes cardiac recovery and reduces the fibrosis of the heart, too. This is due to the increased collagen deposition owing to TGF-β1 factor activation, which, in contrast, is inhibited by the PGE2/EP4 axis [227]. It has been noted that 15-PGDH is overexpressed after coronary artery stent implantation in patients affected by vessel stenosis, lowering the inflammation [228].

#### 5.3.7. Prostaglandin E2 Receptors

As previously stated, PGE2 exerts its functions through four different prostaglandin E2 receptor subtypes: EP1, EP2, EP3, and EP4. These receptors induce distinct effects, mainly based on cell type, and are highly involved not only in disease pathogenesis but even in protection. Each receptor is implicated in several pathological or beneficial conditions. In particular, EP1 has been associated with hypertension and cardiac fibrosis; EP2 with vasodilatation, stimulation of inflammation, and cardiac hypertrophy; EP3 with pro-inflammatory responses, atherothrombosis, hypertension, and cardiac dysfunction; and EP4 with vasodilatation, inflammation, cardiac protection, and atherosclerosis [144,145,147].

They are encoded by four genes, which are *PTGER1*, *PTGER2*, *PTGER3*, and *PTGER4*. These genes present polymorphisms that are associated with several diseases. For instance, polymorphisms in all these genes have been related to hypersensitivity to aspirin [229,230,231,232]. The rs2241360 polymorphism in *PTGER1* and the rs7533733 variant in *PTGER3* have been associated with risk of nephrosclerosis and consequent cardiovascular diseases; specifically, the T allele of rs2241360 (*PTGER1*) is protective against nephrosclerosis and CVD, while the GG genotype of rs7533733 (*PTGER3*) increases the risk and lowers the survival of carriers [233]. A haplotype spanning *PTGER3* (rs2206344, rs3765894, SNP_A-4228934, rs2744918, rs2268062) was associated with hypertension and it is the most common haplotype (ATAAA) that increases the risk of hypertension [234].

A study has identified a haplotype in *PTGER2* as related to a minor risk of myocardial infarction [70]. Another variant in *PTGER2* (rs708494, one of the three SNPs of the haplotype in [70]) would seem to be involved in the development and stability of coronary artery disease [235].

### 5.4. Thromboxane A2 (TxA2)

**Figure 7:**
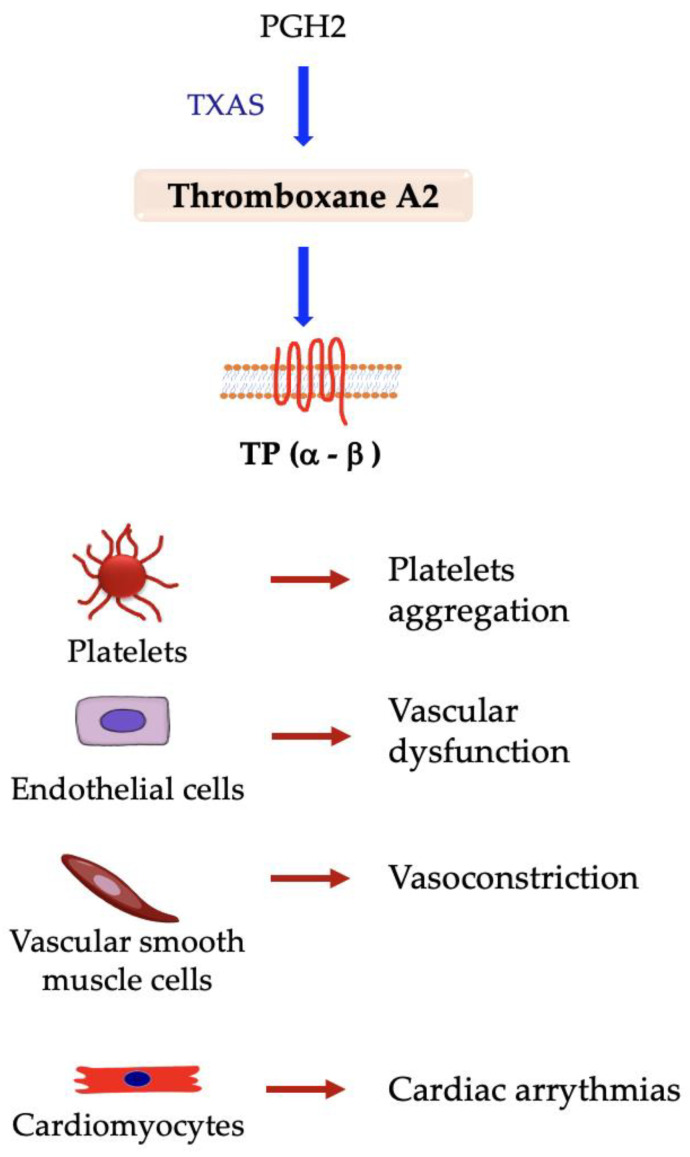
Responses elicited in different cell types by interaction between thromboxane A2 and TP receptor isoforms. → denotes induction.

As the other prostanoids, thromboxane A2 is synthesized from PGH2 by thromboxane A synthase 1 (TXAS) and is rapidly degraded into biologically inactive thromboxane B2 due to its highly unstable nature. TxA2 is mainly produced in platelets and endothelial cells, and its synthesis is generally coupled to COX-1 activity [52]. It is best known as a pro-thrombotic factor, inducing platelet aggregation and thrombosis, and as a vasoconstrictive factor [236,237,238]. TxA2 activity is mediated by the thromboxane prostanoid (TP) receptor, which presents two different isoforms, TPα and TPβ, of which the latter is mostly expressed in endothelial cells [239]. When interacting with TPα on platelets, TxA2 stimulates the activation of these cells, which finally leads to thrombosis owing to cellular shape change, degranulation of pro-thrombotic factors, and platelet aggregation [240]. This thrombotic process normally occurs in primary hemostasis, in which platelets make contact with TxA2 and other pro-thrombotic factors accumulated in the vessel injury site [241]. In inflammatory conditions, such as atherosclerosis, TxA2 synthesis and secretion are increased by pro-inflammatory stimuli, augmenting the likelihood of pathological thrombosis and the risk of coronary artery disease, myocardial infarction, and stroke due to vessel occlusion (Figure 7) [9,242,243,244,245].

It has been demonstrated that, in atherosclerotic plaques, TxA2 synthesis is boosted by activation of PLA2s by pro-inflammatory stimuli [15,38] and even by overexpression of thromboxane A synthase 1. It participates in atherosclerotic lesion formation and in atherothrombosis [246]. In fact, in addition to platelet activation, TxA2 synthesis influences the expression of vascular endothelial cells adhesion molecules ICAM-1, VCAM-1, ECAM-1, and PECAM-1 in these cells, triggering endothelial dysfunction, leukocyte infiltration, and platelet adhesion to the vascular endothelium [247,248]; this promotes atherosclerotic lesions and atherothrombosis pathogenesis. In contrast, TP receptor deletion prevents platelet aggregation, delays the development of atherosclerotic lesions, promotes endothelium integrity, and reduces leukocyte adhesion to endothelium [248].

The thrombotic process is also worsened by TxA2 vasoconstrictive activity on vascular smooth muscle cells. It has been demonstrated that it contributes to the development of angiotensin-II induced hypertension and cardiac hypertrophy [249,250]. The TP receptor mediates hypertension on VSMCs, and its deletion limits the pathogenesis of hypertension [250]; additionally, TP^−/−^ knockout mice have low basal blood pressure [251]. TxA2 exerts vasoconstriction even in the absence of angiotensin-II and, of note, the TP receptor mediates sudden cardiac death following acute injection of a TP agonist, which mimics TxA2 activity [252].

As previously mentioned, TxA2 is involved in cardiac inflammatory tachycardia together with PGF2α [119]. During myocardial ischemia/reperfusion injury, there is an increase in TxA2 levels that impacts the thrombotic risk [253,254]. It has been discovered that the interaction between TxA2 and TP receptors on cardiomyocytes promotes cardiac arrhythmia [255] by eliciting an increase in intracellular Ca^2+^, which influences the electric activity of cardiomyocytes [256]. Moreover, the TxA2-induced Ca^2+^ increase promotes cardiomyocyte cell death [257].

The risk of some cardiovascular diseases decreases along with TxA2 and TP receptor function. Nonetheless, it has been shown that a defective TP receptor or its deletion is related to prolonged bleeding and reduced thrombus formation [251,258]. Overall, TxA2 has a detrimental role in cardiovascular health.

#### Thromboxane A Synthase 1 (TXAS) and TP Receptor

The thromboxane A synthase 1 is encoded by the *TBXAS1* gene, which is highly expressed in platelets and endothelial cells [48], while the TP receptor is encoded by the *TBXA2R* gene. Both TXAS and TP receptors have been linked to cardiovascular diseases, and their levels are elevated in several pathologies [259,260]. It has been shown that TXAS is overexpressed in atherosclerotic plaques [246,261] and even in endothelial cells of hypertensive rats, augmenting the risk of atherothrombosis [262]. During atherosclerosis, the TXAS overexpression is accompanied by upregulation of the TP receptor gene, and their levels increase with the progression of the disease [261]. As would be expected, both TXAS and TP inhibition are associated with reduced risk of thrombosis, atherosclerosis, and hypertension [263].

Several studies have found polymorphisms in *TBXAS1* related to CVDs. For instance, a rare splice variant (rs6962291, TT genotype) in *TBXAS1* was identified as protective against aspirin intolerance in asthmatic patients, since it may give rise to a nonfunctional isoform. It therefore reduces the risk of developing cardiovascular diseases [264]. Another study has identified variants that increase the risk of non-fatal myocardial infarction (rs4725563, rs17181314, and rs3801150) and SNPs that reduce the same risk (rs2267682 and rs8192859) in white individuals [72]. Furthermore, the T allele of rs3801150 was recently associated with stroke risk in the Han Chinese population [265]. In contrast, the rs2267682 polymorphism, which was shown to lower the risk of myocardial infarction [72], has been identified as a risk variant for ischemic stroke in the Northern Han Chinese population [266], likely indicating the influence of different ancestries. Even in the Chinese population, the TT genotype of the rs41708 SNP has been associated with increased risk of atherothrombotic stroke [267,268] and with a higher risk of platelet aggregation [269].

Variants in *TBXA2R* have been associated with bleeding and a lower risk of thrombosis [270]. The rs1131882 in *TBXA2R* has been related to higher platelet activation [268] and has been detected as a risk factor for aspirin resistance in patients affected by ischemic stroke [271]. However, a recent meta-analysis has not confirmed this association, while displaying that rs768963 is linked to a higher risk of stroke [272]. *TBXA2R*−4684T > C and 924C > T polymorphisms were related to aspirin intolerance [273,274,275], and it seems that the 4684T allele lessens the TP receptor expression [273].

### 5.5. Prostacyclin PGI2

Prostacyclin is synthesized by prostacyclin synthase (PGIS) using PGH2 as substrate. Its synthesis is mainly coupled to COX-2 activity [52] and stimulated by pro-inflammatory stimuli. It exerts its function by interacting with the prostacyclin (IP) receptor, and it is produced in many tissues. The principal role of PGI2 is the maintenance of vascular homeostasis, which has anti-inflammatory functions [276,277]. PGI2 plays a critical role in cardiovascular health and counteracts not only TxA2 activity but also that of other prostaglandins. PGI2 promotes vasodilatation, inhibits platelet aggregation, and delays the pathogenesis of atherosclerosis, lowering the risk of developing other cardiovascular diseases, such as myocardial infarction and stroke [276,277]. The beneficial role of PGI2 on the cardiovascular system has been widely demonstrated by several studies via IP receptor inhibition [278]. Furthermore, selective pharmacological inhibition of COX2 enhances vascular thrombosis in mice owing to PGI2 absence [54,55,56].

**Figure 8:**
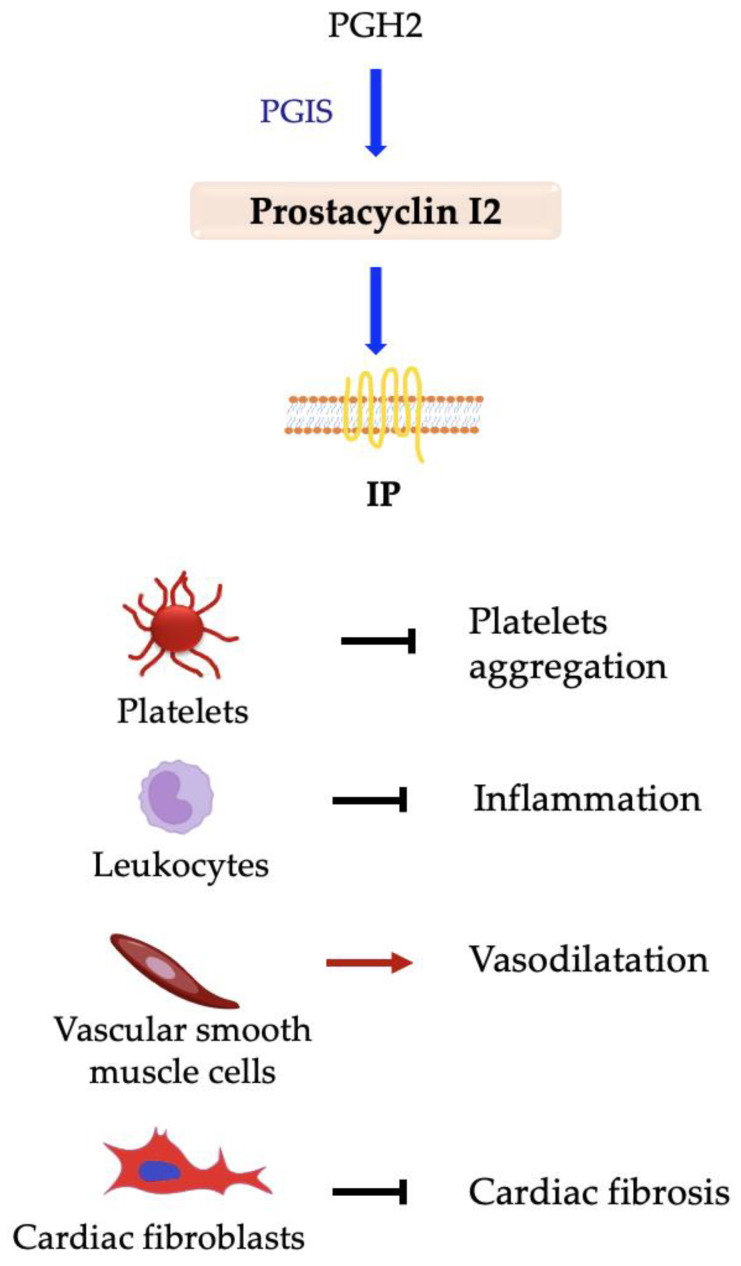
Responses elicited in different cell types by interaction between prostacyclin I2 and IP receptor. → denotes induction; ⊣ denotes inhibition.

A critical role of PGI2 is the inhibition of platelet aggregation, thus reducing thrombus formation and counteracting TxA2 activity. When interacting with the IP receptor on platelets, it determines the expression of sirtuin-1, which in turn inhibits the secretion of pro-thrombotic tissue factor (TF) [55]. It has recently been demonstrated that COX-1 triggers the synthesis of prostacyclin in endothelial cells of healthy mice, contributing to the maintenance of antithrombotic tone (Figure 8) [279].

PGI2 is involved in the resolution of inflammation by blocking the secretion of some pro-inflammatory cytokines in a dose-dependent manner in macrophages [280,281], CD4^+^ T cells [282], dendritic cells [283], and endothelial cells [284]. In addition, it seems that PGI2 acts in synergy with IL-4 and IL-13 to release anti-inflammatory IL-10 from peripheral blood mononuclear cells [285]. A recent study has shown that pro-inflammatory stimuli directly promote the expression of prostacyclin synthase in peripheral macrophages, and the produced PGI2 acts on the same cells, inducing IL-10 secretion and inhibiting pro-inflammatory IL-1β [281]. It helps to prevent the development of atherosclerosis due to its anti-inflammatory properties. It has been shown that PGI2’s beneficial role is reversed by IP receptor deletion. This deletion enhances platelet aggregation and the development of atherosclerotic lesions, impairs the integrity of the endothelium, and increases the leukocytes’ adhesion to the endothelium [248]. IP deletion, combined with acute inflammation, causes increased pain perception in mice and greater thrombi formation after exposition to ferric chloride, which determines endothelium injury; mostly, prolonged exposition to ferric chloride leads to embolic stroke, up to mouse death [286]. Thus, IP deletion in the presence of inflammatory conditions has a detrimental effect on the cardiovascular system.

PGI2 is also involved in the regulation of blood pressure, promoting a reduction of pressure and exerting a protective effect on cardiac function. Indeed, IP deletion is associated with salt-sensitive hypertension and consequent cardiac hypertrophy and fibrosis, increasing the risk of myocardial infarction and stroke [287,288]. PGI2 likely reduces cardiac fibrosis through activation of the PPAR-α receptor [289], which reduces collagen deposition and TGF-β1 levels in cardiac fibroblasts [290]. Furthermore, PGI2 produced by cardiac fibroblasts in response to angiotensin-II directly inhibits collagen synthesis and proliferation of these cells [291]. Going back to hypertension, it has been shown that PGI2 induces rapid pulmonary vasodilatation through activation of PPAR-β, lowering the risk of hypertension [292].

#### PGI2 Synthase and IP Receptor

Prostacyclin synthase is encoded by the *PTGIS* gene and is highly expressed in endothelial cells, but even in many other cells such as macrophages and in the heart [48,293,294]. It has been discovered that PGIS levels increase in the early phases of atherosclerosis, opposing its pathogenesis, but decrease with the progression of atherogenesis, thus facilitating plaque expansion and instability [261]. It is also overexpressed in endothelial cells of hypertensive rats, counteracting the vasoconstriction [262]. PGIS deficiency in mice causes the rise of PGE2 and TxA2 levels, higher blood pressure than wild-type mice, renal fibrosis and necrotic lesions, arterial thickening, and atherosclerosis pathogenesis. All these changes lead to renal infarction and likely increase the risk of myocardial infarction [295]. In contrast, transfection of PGIS in rat arterial endothelium reduces neointima formation after balloon injury in the carotid artery [296] and apoptosis of endothelial cells, promotes vascular angiogenesis, and maintains the vascular tone during hypoxia [297]. Furthermore, overexpression of prostacyclin synthase in the lung reduces pulmonary hypertension, limiting vascular remodelling and vasoconstriction under chronic hypoxic conditions [298]. It has been shown that patients affected by chronic pulmonary hypertension have lower expression of PGIS [299].

Many polymorphisms in the *PTGIS* gene have been associated with cardiovascular diseases. Some polymorphisms and their haplotypes in the promoter region of the *PTGIS* gene have been identified as affecting its expression [300,301] and they might influence the pathogenesis of pulmonary hypertension [302]. A haplotype constituted by seven variants in *PTGIS* (rs561, rs5602, rs729824, rs6090996, rs5628, rs6091000, and rs476496) shows to augment the risk of myocardial infarction in white individuals [72], and among these variants, rs5602 SNP has even been associated with a greater risk of developing atherothrombotic stroke in Chinese [267,268]. Moreover, the CC genotype of rs5629 is related to higher platelet aggregation [269] and is likely a risk factor for myocardial infarction [302].

The prostacyclin receptor is encoded by the *PTGIR* gene, whose expression increases in the presence of inflammatory stimuli [303]. As has been widely demonstrated, the deletion/inhibition of this receptor augments the risk for the pathogenesis of cardiovascular disease. Two synonymous substitutions in *PTGIR* (Val53Val/Ser328Ser) have been associated with enhanced platelet activation and aggregation and a non-synonymous defective variant (Arg212Cys) with hyperplasia of the intima-media in patients affected by deep vein thrombosis [304]. Another study has found that some non-synonymous substitutions (Leu104Arg, Met113Thr, Arg212His, Arg212Cys, Arg279Cys) decrease receptor activity and correlate with a higher risk of coronary artery disease [305].

## 6. Hemiketals

In the last few decades, the hemiketal eicosanoids have been discovered as a new class of eicosanoids derived from arachidonic acid, which seem to play a beneficial role in the cardiovascular system. Hemiketals are produced thanks to a crossover of lipoxygenases and cyclooxygenase pathways in leukotrienes [306,307,308]. Specifically, the COX-2 enzyme is able to use the 5*S*-hydroperoxyeicosatetraenoic acid (5 *S*-HETE) derived from 5-lypoxygenases (5-LOX) activity as a substrate and oxygenate it, giving rise to a diendoperoxide [306,307,308]. The highly instable diendoperoxide is then spontaneously converted to hemiketal D2 (HKD2) and hemiketal E2 (HKE2), which respectively show structural similarities to PGD2 and PGE2, but they play a different role. In addition, H-PGDS is able to catalyze the conversion of the diendoperoxide into HKD2. Anyway, HKE2 is the major product of the non-enzymatic reaction [308].

There are very few studies examining the synthesis and the role of hemiketals. The evidence shows that HKD2 and HKE2 are produced by activated leukocytes, both in vitro and ex vivo, and might act as autocrine or paracrine mediators [308,309,310]. It also would seem that hemiketals are an early product of the inflammatory response [309]. Both HKD2 and HKE2 induce the tubulogenesis of the endothelial cells to form capillary-like structures in a dose-dependent manner [308]. HKE2 is not able to induce the release of cytokines from THP-1 monocytes and differentiated macrophages; thus, it does not foster inflammation [310]. Of note, it has been shown that HKE2 inhibits platelet aggregation [311]. Thus, it appears that HKE2 plays a role comparable to that of PGI2 and has a beneficial role in the cardiovascular system. However, the molecular mechanisms of action of hemiketals have yet to be elucidated.

In addition to hemiketals, the diendoperoxide produced by the crossover between 5-LOX and COX-2, is even converted into hydroxy-prostaglandin D2 (5-OH-PGD2) and hydroxy-prostaglandin E2 (5-OH-PGE2) in human activated leukocytes. As in HKD2 synthesis, H-PGDS uses the diendoperoxide to produce 5-OH-PGD2 [312]. However, neither 5-OH-PGD2 nor 5-OH-PGE2 interact with PGD2 and PGE2 receptors, likely indicating a different role that has not yet been uncovered [312].

## 7. Conclusions

Prostanoids play a critical role in cardiovascular homeostasis and inflammatory processes against pathogens and injury, fostering both pro-inflammatory responses and the resolution of inflammation. In addition, prostanoids are involved in blood pressure regulation and primary hemostasis. As widely stated, they are synthesized in many cell types and perform their function by interacting with specific receptors on cellular membranes. Many studies have highlighted that the expression of prostanoids in different inflammation phases and the interaction between different receptors give rise to distinct pro-inflammatory or anti-inflammatory responses [313]. Therefore, the resolution of inflammation is due to the equilibrium between prostanoids’ synthesis and activity, and their unbalancing is involved in the failure of the resolution of inflammation. This failure determines the switch to chronic inflammation, which alters cardiovascular homeostasis and facilitates the pathogenesis of cardiovascular diseases. For this reason, several anti-inflammatory drugs have been developed to lower inflammation, but some of them increase the risk of thrombosis [49,54,55,56]. Thus, dysfunctional synthesis and activity of prostanoids are involved in the development of many cardiovascular diseases, such as atherosclerosis, hypertension, coronary artery disease, thrombosis, and myocardial infarction. Many studies have also shown that there are genetic variants associated with CVD pathogenesis that alter the expression and catalytic activity of prostanoid synthases and receptors. However, there are sometimes conflicting data, possibly indicating a different influence of geographical ancestries on gene variants.

Finally, prostanoids have been widely studied, and their roles in the cardiovascular system have been extensively elucidated. Nonetheless, there is some conflicting data regarding prostanoids’ activity and their involvement in CVDs as risk or protective factors. It is of paramount importance to note that these contrasting data, concerning the effect of each prostanoid on the same organs and even on cell type might depend on combinations of eicosanoids’ activities and on molecular interactions not yet discovered. To discover this, it is necessary to perform studies which consider more than one prostanoid and under different experimental conditions. It will also be interesting to explore the recently discovered hemiketals and hydroxy-prostaglandins, whose functions we know very little about. Therefore, another effort is needed to fully understand the molecular mechanisms of action of these molecules in order to allow the development of improved therapeutic approaches.

## Figures and Tables

**Table 1:** Signaling pathways of prostanoids.

Prostanoid	Receptor	G-Protein	Signaling Pathway
PGD2	DP1	G_αs_	AC and PKA activation—cAMP increase
	CRTH2	G_αi_	cAMP decrease—Ca^2+^ increase
PGF2α	FP	G_αq_	IP3 and Ca^2+^ increase
		G_α12/13_	Rho activation
PGE2	EP1	G_αq_	IP3 and Ca^2+^ increase
	EP2	G_αs_	AC and PKA activation—cAMP increase
	EP3	G_αi_ or G_α12_	Ca^2+^ increase—Rho activation
	EP4	G_αs_	AC and PKA activation—cAMP increase
TxA2	TPα-TPβ	G_αq_	IP3 and Ca^2+^ increase
		G_α12/13_	Rho activation
PGI2	IP	G_αs_	AC and PKA activation—cAMP increase

Note. AC: adenylyl cyclase. cAMP: cyclic adenosine monophosphate. IP3: inositol trisphosphate. PKA: protein kinase A/cAMP-dependent protein kinase.

## Abbreviations

AA	Arachidonic acid
15-PGDH	15-hydroxyprostaglandin dehydrogenase/Prostaglandin Dehydrogenase 1
15d-PGJ2	15-Deoxy-∆-12,14-prostaglandin J2
5-LOX	5-lipoxygenases
5 *S*-HETE	5*S*-hydroperoxyeicosatetraenoic acid
5-OH-PGD2	5-hydroxy-prostaglandin D2
5-OH-PGE2	5-hydroxy-prostaglandin E2
AC	Adenylyl cyclase
AMI	Acute myocardial infarction
ARIC	Atherosclerosis Risk in Community
CAD	Coronary artery disease
cAMP	Cyclic adenosine monophosphate
COX	Cyclooxygenase
cPGES	Cytosolic PGE2 synthase
CREB	cAMP response element-binding protein
CRP	C-reactive protein
CRTH2	Chemoattractant receptor-homologous molecule expressed on Th2 lymphocytes
CVD	Cardiovascular disease
DP1	PGD2 receptor subtype 1
EETs	Epoxyeicosatrienoic acids
EP	E-type prostanoid receptor
ER	Endoplasmic reticulum
FP	Prostaglandin F receptor
H-PGDS	Hematopoietic prostaglandin D synthase
HETEs	Hydroxyeicosatetraenoic acids
HKD2	Hemiketal D2
HKE2	Hemiketal E2
I/R	Ischemia/reperfusion
IL	Interleukin
IP	Prostacyclin receptor
IP3	Inositol trisphosphate
L-PGDS	Lipocalin-type prostaglandin D synthase
MACE	Major adverse cardiac events
mPGES	Microsomal PGE2 synthase
mPTP	Mitochondrial permeability transition pore
NF-κB	Nuclear factor kappa-light-chain-enhancer of activated B cells
NSAID	Non-steroidal anti-inflammatory drug
PGD2	Prostaglandin D2
PGE2	Prostaglandin E2
PGF2α	Prostaglandin F2α
PGFS	Prostaglandin F synthase
PGG2	Prostaglandin G2
PGH2	Prostaglandin H2
PGI2	Prostacyclin I2
PGIS	Prostacyclin synthase
PKA	Protein kinase A/cAMP-dependent protein kinase
PLA2	Phospholipase A2
PPAR	Peroxisome proliferator-activated receptor
PUFA	Polyunsaturated fatty acid
ROS	Reactive oxygen species
SNPs	Single nucleotide polymorphisms
TF	Tissue factor
TGF-β	Transforming growth factor β
Th	T-helper cells
TNF-α	Tumor necrosis factor α
TP	Thromboxane prostanoid receptor
TxA2	Thromboxane A2
TXAS	Thromboxane A synthase 1
VSMC	Vascular smooth muscle cell
